# Medication errors in the Middle East countries: A systematic review of the literature

**DOI:** 10.1007/s00228-012-1435-y

**Published:** 2012-10-23

**Authors:** Zayed Alsulami, Sharon Conroy, Imti Choonara

**Affiliations:** Academic Division of Child Health, School of Graduate Entry Medicine and Health, University of Nottingham, Derbyshire Children’s at the Royal Derby Hospital, Uttoxeter Road, Derby, DE22 3DT UK

**Keywords:** Medication error, Middle East countries, Prescribing, Administration, Transcribing

## Abstract

**Background:**

Medication errors are a significant global concern and can cause serious medical consequences for patients. Little is known about medication errors in Middle Eastern countries. The objectives of this systematic review were to review studies of the incidence and types of medication errors in Middle Eastern countries and to identify the main contributory factors involved.

**Methods:**

A systematic review of the literature related to medication errors in Middle Eastern countries was conducted in October 2011 using the following databases: Embase, Medline, Pubmed, the British Nursing Index and the Cumulative Index to Nursing & Allied Health Literature. The search strategy included all ages and languages. Inclusion criteria were that the studies assessed or discussed the incidence of medication errors and contributory factors to medication errors during the medication treatment process in adults or in children.

**Results:**

Forty-five studies from 10 of the 15 Middle Eastern countries met the inclusion criteria. Nine (20 %) studies focused on medication errors in paediatric patients. Twenty-one focused on prescribing errors, 11 measured administration errors, 12 were interventional studies and one assessed transcribing errors. Dispensing and documentation errors were inadequately evaluated. Error rates varied from 7.1 % to 90.5 % for prescribing and from 9.4 % to 80 % for administration. The most common types of prescribing errors reported were incorrect dose (with an incidence rate from 0.15 % to 34.8 % of prescriptions), wrong frequency and wrong strength. Computerised physician rder entry and clinical pharmacist input were the main interventions evaluated. Poor knowledge of medicines was identified as a contributory factor for errors by both doctors (prescribers) and nurses (when administering drugs). Most studies did not assess the clinical severity of the medication errors.

**Conclusion:**

Studies related to medication errors in the Middle Eastern countries were relatively few in number and of poor quality. Educational programmes on drug therapy for doctors and nurses are urgently needed.

## Introduction

The Middle Eastern region is strategically, politically and economically important for the whole world. There are fifteen countries between western Asia and northern Africa which make up the Middle Eastern region [1]. Economically, Middle Eastern countries are ranked by the World Bank according to their gross domestic product (GDP) on purchasing power parity (PPP) per capita. The high-income countries (HIC) include Qatar, the United Arab Emirates (U.A.E), Bahrain, Saudi Arabia, Kuwait, Israel and Oman. The upper-middle income countries (UMIC) are Iran, Jordan and Lebanon. The lower-middle iome countries (LMIC) are Egypt, Palestine, Syria, Yemen and Iraq [1]. The population of the Middle Eastern countries is approximately 300 million, with a population growth rate of 1.86 %. Elderly people of 65 years or over represent 3.63 % of the total population of Middle Eastern people [2]. The International Diabetes Foundation estimates that 26.6 million adults (8.6 % of the population) in the Middle East and North Africa currently have diabetes [3]. Obesity rates in the Middle East and North Africa are also among the highest in the world, particularly in the Gulf countries.

Medication errors (MEs) are under-reported in all countries [4], particularly in developing countries. MEs present a universal problem and can cause serious consequences for patients, especially those with acute complex medical conditions [5]. The National Patient Safety Agency revealed that MEs in all care settings in the UK occurred in each stage of the medication treatment process, with 16 % in prescribing, 18 % in dispensing and 50 % in administration of drugs [6].

For paediatric MEs it has been estimated that 3–37 % occur during prescribing, 5–58 % during dispensing, 72–75 % during administration, and 17–21 % are documentation errors [7]. Over an 8-year period in the UK, at least 29 children died due to MEs [8]. Safe drug therapy for children is a major issue in many low-income countries (LIC) and LMIC in the south [9].

Most of the research on MEs has been conducted in HIC in the USA and Europe. Information on the incidence of MEs in LIC and LMIC is limited. In our current research, we have focused on Middle Eastern countries in order to explore and highlight the problem of MEs in this region. There are a variety of reasons why MEs may be different in this region. These include the training of health professionals in clinical pharmacology, differences in relation to the role of clinical pharmacists, and the types of medicines prescribed, alongside cultural issues.

This systematic literature review therefore aimed to identify and review studies of the incidence and types of MEs in Middle Eastern countries, and identify the main contributing factors.

## Methods

### Search strategy

A systematic review of literature relating to MEs in prescribing, transcribing, dispensing, administration and documentation in adults and children in Middle Eastern countries was conducted in October 2011. The following electronic databases were searched: Embase (1980 – October 2011), Medline (1948 – October 2011), Pubmed (until October 2011), the British Nursing Index (1985 – October 2011) and the Cumulative Index to Nursing & Allied Health Literature (CINAHL) (1982 – October 2011). The search strategy included all ages, all languages, and all types of trials and studies. References from eligible articles were also hand-searched in order to identify additional relevant papers.

### Search terms

The following keywords were used as search terms: medication error(s), prescribing error(s), dispensing error(s), administration error(s), documentation error(s), transcribing error(s), medication mistake(s), drug mistake(s), prescribing mistake(s), dispensing mistake(s), administration mistake(s), transcribing mistake (s), wrong medication, wrong drug(s), wrong dose(s), wrong route of administration, wrong calculation(s), physician(s), pharmacist(s) and nurse(s). Each of these key words were combined using “OR” then combined using “AND” with Middle East and also with the names of the appropriate countries (15 countries).

### Review procedure

From previous systematic reviews of MEs, studies have been found to be heterogeneous, as they were conducted in different countries used different definitions and different methods to collect data [10, 11]. For this reason we did not try to analyse the data from a statistical viewpoint, but the results are summarised according to the type of MEs.

### Inclusion/exclusion criteria

We included all types of studies, i.e., randomised controlled trials, non-randomised controlled trials, longitudinal studies, cohort or case–control studies, and descriptive studies that reported the incidence of medication errors or identified the causes of MEs in the Middle East countries, either in adults or children. We excluded reviews, letters, conference papers, opinions, reports or editorial papers.

### Quality assessment

A quality assessment of the identified studies was performed. All relevant studies were reviewed according to 12 criteria adapted from two previous studies [11, 12]. The criteria were adapted to apply to any type of MEs study. Additionally, we evaluated or assessed the documented ethical approval obtained for each study. We therefore evaluated the papers according to the following 13 criteria:

1. Aims/objectives of the study clearly stated.
2. Definition of what constitutes a medication error.
3. Error categories specified.
4. Error categories defined.
5. Presence of a clearly defined denominator.
6. Data collection method described clearly.
7. Setting in which study conducted described.
8. Sampling and calculation of sample size described.
9. Reliability measures.
10. Measures in place to ensure that results are valid.
11. Limitations of study listed.
12. Mention of any assumptions made.
13. Ethical approval.

## Results

### Search results

**Fig. 1:**
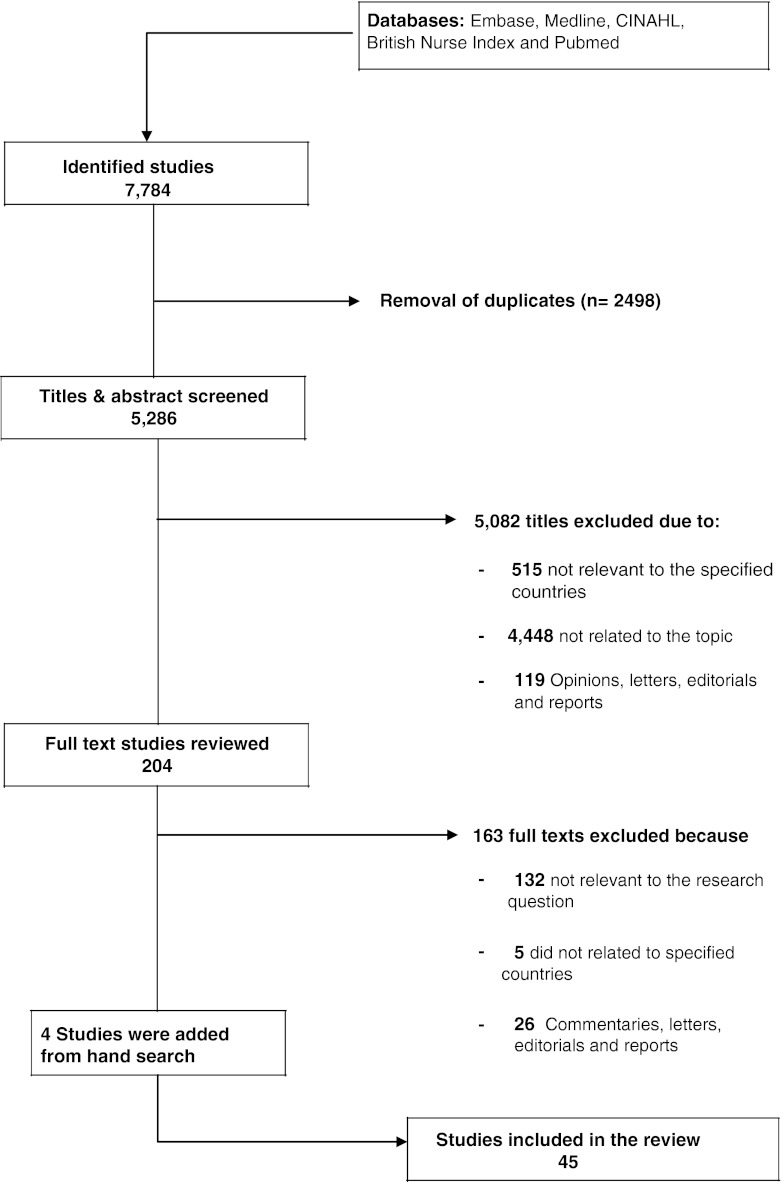
Flow chart for search and review process

The results of this search strategy can be found in Fig. 1. More than 5,000 articles were excluded, as the papers were either not related to the specified countries or not relevant to MEs. This left 204 articles for full-text review. A further 163 articles were excluded because they were not relevant to the topic, not related to the specified countries or were opinion articles, letters, editorials and reports.

Forty-one articles were identified as relevant. Four additional relevant studies were identified after hand-searching of the references of these studies. Forty-five articles were therefore finally relevant and are included in this systematic review (Fig. 1). The abstracts of four studies were in English but the full texts were in foreign languages (3 in Persian and 1 in Hebrew), and those papers were translated into the English language.

### Countries with data

**Fig. 2:**
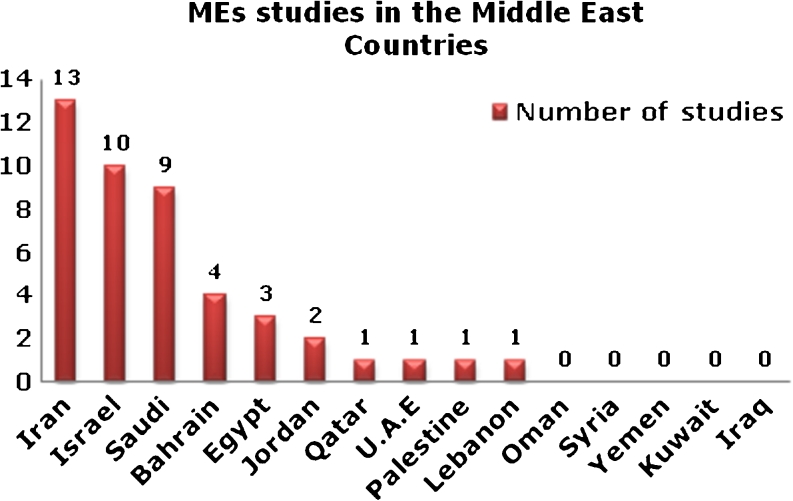
Graph illustrating origin of included studies

The 45 studies provided data for 10 of the 15 countries of the traditional Middle East (Fig. 2). These included 13 studies in Iran, 10 studies in Israel and 9 studies in Saudi Arabia. There was no available data on MEs in the following countries: Yemen, Kuwait, Iraq, Oman and Syria.

### Quality assessment of studies

**Fig. 3:**
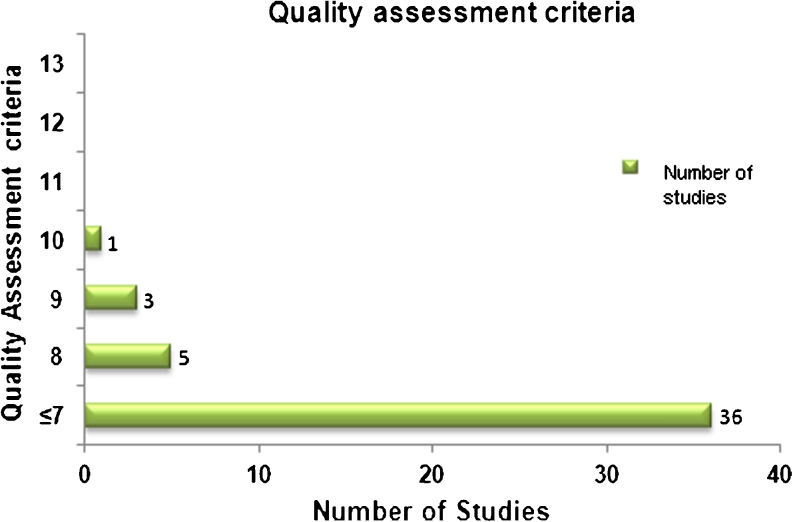
Quality assessment criteria of included studies

After the application of the quality assessment criteria which were modified to apply to any type of MEs study, no study met all the 13 criteria. Only one study fulfilled 10 criteria, three studies met 9 criteria, and five met 8 criteria. The remaining studies met less than 7 criteria (Fig. 3). Ten of the 45 studies did not specify the type of MEs and 14 of the 45 studies did not clearly state whether or not ethical approval was obtained.

### Types of medication error studies

Twenty-one of the 45 studies assessed prescribing errors (Table 1). Most (seven) of these studies were conducted in Saudi Arabia and in Israel (five). One study assessed transcribing errors (Table 2). Eleven studies measured medication administration errors (Table 3) and most of these studies were performed in Iran. Tables 4 and 5 contain the 12 intervention studies that have been performed in Middle Eastern countries for adults and paediatric patients. Four of these studies were conducted in Israel. No studies were identified which evaluated dispensing errors and documentation errors in Middle East countries. Nine (20 %) studies out of the 45 studies focused on MEs in paediatric patients.

**Table 1:** Studies describing prescribing errors

Country (Setting)	Type of study	Duration	Sample	Outcome	Reference
Israel (General hospital)	Prospective, prescriptions were reviewed in pharmacy.	6 months	14,385 prescriptions	160 MEs were detected; 97 (60.6 %) were prescribing errors; Incorrect dosage (44) was the most common type.	[14]
Bahrain (Primary care)	Prospective, prescriptions were collected by pharmacists.	2 weeks	77,511 prescriptions	7.7 % of prescriptions contained errors; Omission errors (93.6 %), Commission errors (6.3 %).	[15]
Iran (Teaching hospital)	Prospective, prescriptions from elderly patients were collected.	4 months	3000 prescriptions	829 (27 %) patients received at least one inappropriate prescription; 746 (24 %) patients had at least one medicine prescribed in duplicate.	[22]
Bahrain (Primary care)	Prospective, prescriptions for infants were collected by pharmacists.	2 weeks	2282 prescriptions	90.5 % of prescriptions contained errors; 74.5 % of medications contained drug errors; Dosing frequency was incorrectly written in 20.8 % and dose strength was incorrectly in 17.7 %.	[16]
Palestine (General hospital)	Prospective, all patients with creatinine clearance ≤59 ml/min were included, data were collected from patients’ files.	4 months	78 patients	63 (80 %) patients were having at least one inappropriate medication; 1.5—fold greater than the recommended dose, poor knowledge of pharmacokinetics of prescribed drug.	[23]
Egypt (Teaching hospital, ICU)	Prospective, direct observation by pharmacist was conducted to record medication-related problems.	1 year	220 patients	619 medication-related problems were detected in 213 patients; Incorrect dosing (22 %) was the most common errors in ICU.	[24]
Bahrain (Primary care)	Prospective, iron prescriptions for infants were collected and reviewed by pharmacist.	2 weeks	2,282 prescriptions	159 prescriptions included iron preparation; 56 out of 159 were issued without dosage forms and duration of therapy.	[17]
Iran (Teaching hospital)	Prospective, drug order sheets in nephrology ward were reviewed by clinical pharmacist.	4 months	76 patients (818 medications)	86 (10.5 %) prescribing errors were detected in 46 of the admissions; wrong frequency (37.2 %), wrong dose (19.8 %) and overdose(12.8 %) were the most common types of errors.	[18]
Saudi (Primary care)	Prospective, prescriptions were reviewed in public and private centres	NR	600 prescriptions	64 (72 %) physicians were classified as writing low-quality prescriptions	[25]
Saudi (Teaching hospital)	Prospective, medication charts and orders data collected by pharmacists	1 month	1582 medication order	113 (7.1 %) prescribing errors were detected; Wrong strength 39 (35 %) followed by wrong dose frequency 26 (23 %).	[26]
Saudi (Primary care)	Prospective, all medication prescriptions were analysed.	1 working day	5299 prescriptions	990 (18.7 %) prescribing errors identified; 8 (0.15 %) prescribing errors had serious effect on the patients.	[27]
Bahrain (Primary care)	Prospective, prescriptions issued by the residents were collected by pharmacists	1 year	2692 prescriptions	2372 (88 %) prescriptions had errors; total number of errors was 7139; Incorrect dose and wrong dose frequency (24.7 %) of errors.	[19]
Israel (Teaching hospital)	Prospective, case–control study	18 months	274 patients	137 MEs were identified; 63 (46 %) errors were prescribing.	[20]
Saudi (Teaching hospital)	Retrospective, all prescriptions obtained from pharmacy were analysed by physicians and pharmacists.	1 year	3796 prescriptions	94 % of prescriptions had no quantity indicated; 90.7 % of prescriptions had incomplete instructions for patients.	[28]
Iran (Paediatric hospital)	Retrospective, hospital information-based study.	6 months	898 medical charts	Incomplete information in 74 % of medication orders; Time of drug administration not reported in 47.8 % of medical charts.	[29]
Israel (Teaching hospital, ED)	Retrospective, charts review was performed by two physicians for adult patients transferred by ambulance.	1 year	471 patient charts	24 (12.7 %) of 188 patients receiving drugs in vehicle were subject to MEs; In ED 120 (36 %) of 332 patients were subject to MEs.	[30]
Saudi (Tertiary hospital, PICU & paediatric wards)	Retrospective of paediatric physicians medication orders.	5 weeks	2,380 medication orders	1,333 MEs were reported; 1,051 (78.8 %) errors were potentially harmful; Incidence rate was 56 per 100 medication order; Dose errors were the highest incidence (22 %).	[21]
Saudi (General Hospital)	Medical records were reviewed for adult patients.	2 years	2627 patient files	3963 MEs were identified; 60 % of patient files contained one error; 30 % of patient files contained two errors, and 3 errors or more found in 10 % of patient files. Wrong strength was reported in 914 patients (35 %).	[31]
Israel (Not applicable)	Questionnaire	NR	N/A	18 % of doctor orders were written according to the hospital standard; 3 % of doctors and 25 % of nurses were the rate of compliance in ED.	[32]
Israel (Not applicable)	Structured questionnaire sent to active physicians to evaluate the rates and types MEs that they had encountered.	NR	627 physicians	470 (79 %) physician made an error in prescribing; 376 (63 %) physicians made more than one error; 94 (16 %) physicians made one error.	[33]
Saudi (Primary care)	Self—administered questionnaire designed to explore factors influencing prescribing.	NR	87 physicians	47 (54 %) physicians believed that limited knowledge of pharmacology is a main cause of prescribing errors; 65 % of physicians had not received training in drug prescribing; 34 % of physicians had consulted a pharmacist before drug prescribing.	[34]

MEs: Medication Errors; NR: Not Reported, N/A: Not Available.

**Table 2:** Study describing transcribing errors

Country (setting)	Type of study	Duration	Sample	Outcome	Reference
Iran (Teaching hospital)	Prospective, direct observational study to detect transcribing MEs.	5 months	287 medication charts	289 errors were identified with average one error per chart; Omission error was rated as the highest (52 %) transcription error found in this study	[35]

MEs: Medication Errors

**Table 3:** Studies describing administration errors

Country (setting)	Type of study	Duration	Sample	Outcome	Reference
Iran (Teaching hospital, ICU)	Prospective, random observational study by pharmacists for preparation and administration of IV drugs by nurses	2 weeks	524 preparation & administration process	380 (9.4 %) errors were identified out of 4040 opportunities for errors; 33.6 % were related to the preparation process; 66.4 % were administration errors; Injection of bolus doses faster than recommended was 43 % of errors.	[38]
Israel (Three hospitals; 32 wards)	Multi-method (observations, interviews, administrative data) were conducted to test the learning mechanisms to limit MAEs.	NR	173 nurses	One patient in 3 was exposed to MAEs each time they received medication.	[39]
Lebanon (10 community pharmacies)	Retrospective, each patient profile was reviewed and to confirm patient record information in-person interviews by qualified pharmacists.	7 months	277 patients	60 % of patients were taking at least 1 inappropriate medication; Missing doses represented 19 % of patients with inappropriate medication .	[41]
Saudi (Teaching hospital)	Retrospective, incident reports documented by physicians and nurses were collected.	2 years	23,957 admissions	38 MEs were reported; Incidence rate of MEs was 1.58 per 1000 admission; Missed medication was the most common error in 15 (39.5 %) patients; 50 % of the errors occurred at night.	[42]
Jordan (24 hospitals)	Descriptive (questionnaire) study of nurses’ perceptions about rate, causes and reporting of MEs.	NR	799 nurses	Average number of MEs per nurse was 2.2; 42.1 % of MEs were reported to nurse managers; 60 % of nurses failed to report MEs.	[43]
Iran (Cardiac care unit)	Questionnaire study to investigate the frequency, type and causes of MEs in cardiac care unit.	NR	60 nursing students	10 % of nursing students had made MAEs; Incorrect drug dose calculation, poor drug knowledge were the most common type of errors.	[44]
Iran (Teaching hospital)	Survey study to investigate the frequency, type and causes of MEs of nursing students	NR	76 nursing students	17 % of nursing students reported MAEs; Wrong drug dose was the common cause of error.	[45]
Iran (Three nursing schools)	Descriptive self—report questionnaires	(Winter 2008)	240 nursing students	124 MAEs were made by student nurses; 0.5 average number of MAEs per nurse.	[46]
Iran (Different hospitals)	Questionnaire study was conducted to identify nursing errors and the related factors	(in 2010)	239 nurses & head nurse	Lack of compiling and reviewing of the drug allergies and medical history of the patient were 34.7 % and 31.7 %, respectively; Lack of considering the proper time of giving the medicines were 27.5 % and 31.7 %, respectively.	[47]
Jordan (Teaching hospital)	Descriptive questionnaire study to determine the types, stages and factors contributing to MEs.	Not reported	126 nurses	Rate of MEs of nurses, physicians and pharmacists were 48 %, 31.7 % and 11.1 %, respectively; Wrong dosage and wrong patient were the highest type of MEs reported.	[40]
Iran (Teaching & non teaching hospitals)	Questionnaire study performed to evaluate the relationship between the incidence and reporting of MEs by nurses and work conditions.	7 months	286 nurses	19.5 ME cases were recalled by each nurse; Relationship between error incidence and nursing work load was statistically significant.	[48]

ICU: Intensive Care Unit; MAEs: Medication Administration Errors; MEs: Medication Errors; NR: Not Reported

**Table 4:** Interventional studies in adults

Country (Setting)	Intervention	Duration	Sample	Outcome	Reference
Israel (Teaching hospital)	Comparison between prescription orders using CDOE and Handwritten order	NR	4600 hospitalization days	Prescribing errors occurred in handwritten orders (11.3 %) higher than in CDOE (3.2 %); The use of CDOE was associated with a significant reduction in mean hospital stay.	[49]
Israel (Teaching hospital)	Comparison between CDOE and handwritten order in similar department	NR	1350 adult patients (641 handwritten,709 CDOE)	Incidence of prescribing errors by handwritten orders (7.2 %) was higher than in CDOE (3 %); CDOE has a large impact on the prevention of prescribing errors.	[50]
U.A.E (Teaching hospital)	Clinical pharmacists established training and educational materials for inpatient nurses about MEs.	4 months	370 nurses	The clinical pharmacist’s program has improved knowledge of the inpatient nursing staff in terms of raising their awareness about medication errors.	[51]
Qatar (4 Primary care)	Pharmacists in four clinics within the service used online, integrated health care software to document all clinical interventions made.	3 months	82,800 patients	589 (0.7 %) patients prescriptions were intercepted for suspected errors; 51 % of the interventions were related to dosing errors.	[52]
Egypt (Teaching hospital, cancer centre)	Clinical pharmacy interventions (detecting MEs, correcting those errors, sending recommendations to medical staff).	1 year	89 adults, 11 paediatrics	MEs reduced from 1548 to 444 errors after clinical pharmacy intervention implemented; 76 % of the errors recorded occurred in the prescribing stage.	[53]
Iran (Teaching hospital, infectious disease ward)	Assessment of the clinical pharmacists role in detecting and preventing of MEs.	1 year	861 patients	112 MEs were detected by clinical pharmacists; physicians were responsible for MEs more than nurses and patients 55 (49 %), 54 (48 %), 3 (2.7 %) respectively. Drug dosing, drug choice were the most common error types.	[54]
Saudi (Three Goverement Centres)	Three types of interventions were evaluated: pre/post improve the quality of physicians prescriptions	NR	61 physicians	In pre: 8 % of prescribed drugs were with major errors. In post: 2 % of prescribed drugs were with errors.	[55]

CDOE (Computerized Drug Order Entry); MEs: Medication Errors; NR: Not Reported; U.A.E: United Arab Emirates.

**Table 5:** Interventional studies in paediatric and neonatal patients

Country(Setting)	Intervention	Duration	Sample	Outcome	Reference
Israel (Children Hospital, PCCD)	Pre/post implementation of CPOE and CDSS	3 years	13124 drug orders in first part 46970 orders in second part	3 errors were identified in the first part of the study (all were overdoses); No errors were identified in the second part.	[56]
Israel (Children hospital, PICU)	CPOE implementation in four different periods	3 years	5000 PICU medication orders	273 (5.5 %) medication orders contained prescription errors; 83 % of prescription errors were reduced after CDSS implemented.	[57]
Iran (Teaching hospital, Neonatal ward)	Comparison between physician order entry (POE) and nurse order entry (NOE) methods effect on reducing dosing MEs.	4 months	158 neonates	80 % of non-intercepted medication errors in POE (period 1) occurred in the prescribing stage compared to 60 % during NOE (period 2); Prescribing errors were decreased from 10.3 % with POE to 4.6 % with NOE period, respectively.	[58]
Iran (Teaching hospital, Neonatal ward)	Comparison of CPOE effect without and with CDSS in three periods.	7.5 months	248 patients	MEs rates before intervention implemented (period 1) was 53 %; Implementation of CPOE without CDSS the MEs rate was 51 %; After CDSS was added the MEs rate was 34 %; Overdose was the most frequent type of errors.	[59]
Egypt (Teaching hospital, PICU)	Pre/post (physician education; new medication chart; physician feedback) study of prescribing errors in PICU.	10 months	Pre :1417 medication orders, Post: 1096 medication orders	1107 (78 %) orders had at least one prescribing error; Significant reduction in prescribing error rate to 35 % post-intervention (P < 0.001); Severe errors reduced from 29.7 % to 7 % after intervention.	[60]

PCCD: Paediatric Clinical Care Department; CPOE: Computerized Physician Order Entry; CDSS: Clinical Decision Support System; PICU: Paediatric Intensive Care Unit.

### Incidence of medication errors

**Fig. 4:**
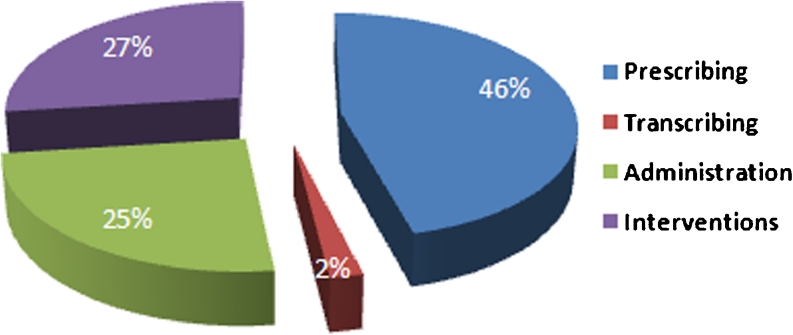
Study classification by stage of medication process

The incidence of MEs in this review is difficult to compare between studies because different methodologies and different definitions were used. We classified our results according to where they occurred during the medication treatment process, i.e., prescribing, transcribing and administration (Fig. 4).

### Prescribing errors

Prescribing errors have been defined as MEs initiated during the prescribing process. These include the incorrect selection of medication, wrong dose, wrong strength, wrong frequency, incorrect route of administration, inadequate instruction for use of a medication and wrong dosage form [13]. Twenty-one (46 %) of the studies reported MEs that occurred during the prescribing stage of the medication process (Table 1). Eight studies identified in this review used the above definition [14–21], while the remaining studies did not clearly state a definition of prescribing errors. Thirteen were prospective studies and were conducted in six countries [14–20, 22–27]. Five were retrospective studies [21, 28–31], and the remaining three studies were questionnaires [32–34]. Four studies assessed prescribing errors in children [16, 17, 21, 29].

Estimates of the results were difficult to compare between studies because rates of error were expressed differently. Al-Khaja et al. reported the highest error rate, which was 90.5 % of prescriptions in a primary health care centre [16], while the lowest rate, reported by Al-Dhawailie, was 7.1 % of prescriptions in a teaching hospital [26]. The most common types of prescribing errors reported among the Middle Eastern countries were incorrect dose, wrong frequency and wrong strength. This systematic review revealed that the percentage of dosing errors that were reported during medication prescribing ranged from 0.15 % to 34.8 % of the prescriptions (Table 6).

**Table 6:** prescriptions with dosing errors

Country (setting)	No. of Prescriptions or medication orders	Dosing errors (number)	Dosing errors (%)	References
Israel (General hospital)	14,385 prescriptions	44 prescriptions	0.3	[14]
Bahrain (Primary care)	77,511 prescriptions	1,413 prescriptions	1.8	[15]
Bahrain (Primary care)	2,282 prescriptions	795 prescriptions	**34.8**	[16]
Egypt (Teaching hospital)	2,286 medication prescribed	503 medication prescribed	22	[24]
Bahrain (Primary care)	2,282 prescriptions	60 prescriptions	2.6	[17]
Iran (Teaching hospital)	86 prescriptions	11 prescriptions	12.8	[18]
Saudi (Primary care)	1,582 medication orders	14 medication orders	0.89	[26]
Saudi (Primary care)	5,299 prescriptions	8 prescriptions	**0.15**	[27]
Bahrain (Primary care)	5880 medication orders	397 medication orders	6.7	[19]
Israel (Teaching hospital)	4736 prescriptions	31 prescriptions	0.65	[20]
Israel (Teaching hospital)	471 medication orders	12 medication orders	2.5	[30]
Saudi (Tertiary hospital)	2,380 medication orders	526 medication orders	22.1	[21]

### Transcribing errors

One prospective study of transcription errors using a direct observational method was performed in Iran (Table 2) [35]. Transcribing errors were defined as any deviation in transcribing a medication order from the previous step. This study used a direct observation method of the transcribing process. The transcribing process involved prescriptions being either transferred to the hospital pharmacy or dispensed from ward-based stocks. When prescriptions were sent to the pharmacy, all the information was transcribed into the pharmacy information system. The pharmacy staff then filled, checked and dispatched the drugs to the ward. The observation process included review of each medication order on the order sheet, its transcription, administration nursing note and documentation of its prescriptions to the pharmacy database. The study result revealed that over 50 % of omission errors occurred at transcription stage.

### Administration errors

Administration errors have been defined as a discrepancy between the drug therapy received by the patient and that intended by the prescriber or according to standard hospital policies and procedures [36, 37]. Three studies used this definition [38–40], while the remaining eight studies mainly used general definitions of MEs rather than a medication administration error definition. Two were prospective studies (Table 3) [38, 39]. One was an observational study in Iran which assessed the administration of intravenous drugs [38]. The other study, in Israel, used a variety of methods (observations, interviews and administrative data) [39]. The variation between the studies in definitions and methods used for data collection made comparisons difficult. The study that defined errors in preparation and administration, found that the error rates were higher in the administration process compared to the preparation process for intravenous medications, and within the administration process the technique of administration of bolus injection was the most common error (43.4 %) [38].

Two were retrospective studies [41, 42]. Seven were questionnaire studies of nurses’ perceptions of administration errors [40, 43–48]. The reported administration error rates ranged from 9.4 % to 80 % [38, 41]. Saab et al. reviewed patient records and confirmed their results through interviews with patients [41]. They found that the use of an inappropriate drug was higher when patients used both over-the-counter (OTC) and prescription medicines [41]. Sadat-Ali and colleagues assessed the prevalence and characteristics of MEs in patients admitted to a teaching hospital [42]. The authors found that the prevalence of MEs was low (1.58 per 1000 admission). This is likely due to the method used in the study, which was a retrospective review of incident reports—notorious for underestimation of error rates [42]. In addition, the authors revealed that most of the MEs (50 %) occurred during the night shift [42]. Seven questionnaire studies were conducted (5 in Iran and 2 in Jordan). All these studies evaluated nurses and student nurses opinion about the drug administration errors in their area of work [40, 43–48] (Table 3).

### Interventional studies

Twelve (27 %) studies were identified describing interventions used to reduce MEs [49–60]. Of these, seven interventions were implemented in adult patients (Table 4) [49–55], and five interventions in paediatric and neonatal patients (Table 5) [56–60]. The interventions had been evaluated in studies from 3 months to 3 years, and most studies involved a comparison of computerised drug order entry systems, with or without clinical decision support systems (CDSS), and/or with handwritten prescriptions. The outcomes for all interventions were positive and led to the prevention and reduction of MEs.

Four interventional studies examined the role of the clinical pharmacist in reducing MEs [51–54]. All these studies were in adult patients only. These interventions led to a significant reduction in the number of MEs. Most of the errors detected were in the prescribing stage. Incorrect drug dosing, incorrect drug choice and drug interactions were the most common errors detected by clinical pharmacists. One of the intervention studies used a self-reported questionnaire designed to collect data after the clinical pharmacists established training and educational materials for inpatient nurses about MEs. No ME data was actually observed or collected [51].

In paediatric and neonatal patients, computerised physician order entry interventions, with and without CDSS, was the most commonly used intervention. All interventions that were implemented in paediatric patients found that medication error rates decreased after the CDSS was added to the computerised physician order entry system [56, 57, 59].

In addition, one study among the interventional studies in paediatric patients was conducted to compare two medication order entry methods: the physician order entry (POE) and nurse order entry [58]. The authors found that the error rates decreased within the NOE period compared to the errors within the POE period [58].

### Types of errors

Incorrect dose was the most common type of error reported in 12 studies [14–21, 24, 26, 27, 30]. The dose error rates were reported from 0.15 % to 34.8 % of prescriptions errors (Table 6). Other studies included wrong frequency errors [16, 17], wrong strength [15, 16, 20, 25, 31], wrong or without dosage form [15, 16, 24, 27] and wrong duration of therapy [15, 16, 24, 27].

### Medications involved

Differences in the reporting of medications between studies were apparent; some studies involved the medication names, and others listed only the therapeutic class. Most of the errors were related to antihistamine drugs [16, 22, 27], antibiotic medications [14, 23, 38, 60] and anticoagulant drugs [45, 54, 60]. In addition, medications reported in studies conducted on paediatric patients found that antihistamines, paracetamol, electrolytes and bronchodilator drugs were the most common drugs associated with errors [16, 30].

### Severity of reported medication errors

The majority of studies did not assess the clinical consequences of reported MEs. Six (13 %, 6/45) attempted to classify the severity of the MEs [14, 21, 26, 30, 31, 38] (Table 7). Only one study reported the severity of the MEs in detail, but was a retrospective study [21]. Two other studies were retrospective [30, 31], while the other three were prospective studies [14, 26, 38]. One study reported 26 deaths and felt that MEs were a contributory factor [31].

**Table 7:** Clinical consequences and medicines of reported medication errors

Country	Type of error	Medicines	Clinical consequences	Reference
Israel	Prescribing	Anti-infectives, TPN, cytotoxics	Errors divided into potentially serious, clinically significant and clinically non-significant. MEs most frequent in haemato-oncology and these were the errors that were of greatest clinical significance	[14]
Saudi Arabia	Prescribing	Not stated	Examples of potentially serious errors were given including tenfold errors of amphotericin and captopril	[26]
Israel	Prescribing	Cardiovascular drugs	14 MEs (8 %) were clinically significant. There were also 3 (2 %) severe MEs	[30]
Saudi Arabia	Prescribing	IV fluids, antibiotics, bronchodilators, opioid analgesics, cardiovascular drugs, sedatives	Majority of MEs were potentially harmful (1051, 79 %)	[21]
Saudi Arabia	Prescribing	Antibiotics, cardiovascular drugs	MEs were a contributory factor to 26 deaths	[31]
Iran	Administration	Antibiotics, antacids, corticosteroids	No clinically significant errors detected	[38]

### Factors contributing to medication errors

The determination of factors contributing to MEs is an important aspect of this review because preventing MEs from reaching the patient depends on a sound knowledge of the causes or contributing factors. The factors contributing to MEs were reported in 12 studies [15, 16, 19, 21, 26, 27, 31, 40, 42, 45–47]. The most common factors reported in this review were as follows:

- Lack of knowledge of prescribing skills.
- Lack of pharmacological knowledge of physicians and nurses.
- Poor compliance with drug prescribing and administration guidelines.
- Lack of reporting of MEs.
- Heavy workload and new staff.
- Miscommunications between health care professionals.

In general, poor knowledge of medicine prescribing and administration was the most common reported contributory factor of MEs in Middle Eastern countries [15, 16, 26, 31, 45, 47].

## Discussion

The aim of this systematic review was to review studies of the incidence and types of MEs in Middle Eastern countries and to identify the main contributory factors involved. MEs are an important variable in determining patient safety. This review showed that there have been relatively few studies of MEs in the Middle East compared to the number from the US and Europe [61]. To our knowledge, no previous systematic review has evaluated MEs in Middle Eastern countries. Additionally, the quality of the studies in the Middle East was poor. Poor knowledge of clinical pharmacology was a major factor in many of the papers. This systematic literature review has shown that the scientific literature on MEs published in Middle Eastern countries is limited. No information was available on five of the countries included in the review. Many studies focused mainly on adult patients.

### Prescribing errors

Many differences were found with regard to how the studies obtained and reported data. Most of the studies in Middle Eastern countries evaluated MEs during the prescribing stage. The reported incidence of prescribing errors in this review ranged from 7.1 % to 90.5 % of medication orders. A high rate of prescribing errors is known to be an international problem [61, 62]. In a previous systematic review conducted in the UK to identify the prevalence, incidence and nature of prescribing errors in hospital inpatients, prescribing errors were found to be a common occurrence [61], and this is consistent with our findings. The incidence of prescribing errors in that review were 2–14 % of medication orders [61], which was lower than that found in our review of MEs in Middle Eastern countries. However, another study in the UK found that prescribing error rates vary widely, ranging from 0.3 % to 39.1 % of medication orders [63]. It is possible that the incidence rate of prescribing errors in the Middle Eastern countries is higher than that reported in other countries in the world, for example in the UK, but it could also be due to methodological differences.

### Transcribing errors

Although some studies classified the transcribing stage as the third most important area in the medication treatment process, Lisby et al. identified the transcribing stage as the area in which most errors occur [64]. In our review only one study assessed transcribing errors, and found that over 50 % of omission errors occurred at transcription stage; this is consistent with other studies [64, 65]. The shortage of studies in this stage of medication treatment may distort the reality of the incidence rate of errors.

### Administration errors

Our review showed that administration errors occurred in 9.4 % to 80 % of drug administrations. This is higher than that reported in studies in HIC. Two observational studies found that medication administration error rates in the acute care setting varied between 14.9 % and 32.4 % in France and Switzerland, respectively [66, 67].

In our review only one observational study determined the frequency of MEs that occurred during the preparation and administration of intravenous drugs in an intensive care unit. It found that the rate of errors in drug administration (66.4 %) was higher than in preparation (33.6 %) [38]. Another study found that the medication administration error rate for intravenous medication is significantly higher than other types of medication, the researchers observing the preparation error rate as 26 % and the administration error rate as 34 % [68]. Our findings therefore are also consistent with the previous studies’ results highlighting that intravenous MEs occur more frequently than preparation errors [68]. In addition, Armitage and Knapman found that the frequency of administration errors ranges from 2.4 % to 47.5 %, depending on the drug distribution system in place [69]. The difference between the results of these studies may be affected by the different definitions and different methods used, and may also be due to the place of research and settings. However, in the UK a recent report by National Patient Safety Agency (NPSA) specified that 56 % of reported errors associated with severe harm occurred at the administration step [70].

Our results indicate that the most common types of errors reported were incorrect drug dose, wrong frequency and wrong strength during the prescribing stage. This is consistent with previous studies’ results. In comparison, studies of MEs in US and UK hospitals found that incorrect doses were the most common type of error [71]. The UK National Patient Safety Agency (2009) reported that the most common type of medication errors that occurred in the NHS was a wrong dose or wrong frequency of medications [6], also consistent with our findings.

Based on our review results, the main factor contributing to MEs in Middle Eastern countries is poor knowledge of medicines in both doctors (prescribers) and nurses (administering drugs), as was also the case with other studies’ [72]. Educational programmes for drug prescribers and nurses concerning drug therapy are urgently needed to avoid drug errors and to improve patient safety by clinical pharmacists and clinical pharmacologists. Different studies have found that clinical pharmacists play a significant role in delivering training and competency assessment [73].

### Limitation of this review

Some limitations of this review should be considered in interpreting the results. The search strategy and search terms were designed in order to be as comprehensive as possible, but the databases used were directly biased to English-language research and studies. We therefore may have missed some studies because the original languages of the included countries of the Middle East is not English; all of the included countries speak Arabic except Iran (Persian) and Israel (Hebrew).

## Conclusion

As the first systematic review to describe MEs in Middle Eastern countries, this review aimed to find out which scientific literature has reported on or evaluated MEs in Middle East countries. Although the studies related to MEs in the Middle Eastern countries were relatively few in number, there was a wide variation between studies in the error rates reported, and this may due to the variations in their definitions of medication errors, settings, the denominators and methodologies used. However, the quality of MEs studies that were identified in this review was poor. Most of the studies were conducted on adult patients, while very few MEs studies have been performed in paediatric hospitals. Many studies focused on prescribing errors and factors contributing to MEs. Our findings highlighted that poor knowledge of medicines was a contributory factor in both prescribers and nurses administering drugs. Middle Eastern countries urgently need to introduce educational programmes to improve prescribing skills and knowledge of prescribers, and to encourage nurses to improve their quality of drug administration.

## Suggested recommendations

According to the review results, the following recommendations are suggested to allow decision-makers to improve medication safety and reduce MEs in Middle Eastern countries:

- Increase the awareness of MEs of health care professionals.
- Prescribers need to pay more attention to drug dosing.
- Improve medication error reporting systems and policy among the Middle Eastern countries by removing barriers, clarifying the importance of reporting and encouraging health care professionals to report medication errors.
- Clinical consequences of MEs should be assessed and evaluated in future studies
- Carry out regular intensive educational and training programmes in pharmacotherapy for undergraduate medical and paramedical students.
- Educational programmes by clinical pharmacists and clinical pharmacologists on drug therapy are urgently needed for doctors and nurses.
